# Progress in diagnosing and treating thyroid squamous cell carcinoma under the 5th edition of WHO classification

**DOI:** 10.3389/fendo.2023.1273472

**Published:** 2024-01-18

**Authors:** Wei Ding, Xiaofan Gao, Xuan Ran

**Affiliations:** Department of Thyroid Surgery, The Second Hospital of Jilin University, Changchun, China

**Keywords:** squamous cell carcinoma, thyroid, primary squamous cell carcinoma, secondary squamous cell carcinoma, anaplastic thyroid carcinoma

## Abstract

Squamous cell carcinoma of the thyroid (SCCT) is a rare thyroid gland malignancy, with only a few hundred cases reported in the literature, mostly as case reports or small sample studies. In the previous WHO classification, squamous cell carcinoma of the thyroid was defined as a carcinoma composed entirely of squamous cells without differentiated carcinoma components. It was once included in the WHO tumor classification separately. However, the 2022 WHO classification of squamous cell carcinoma of the thyroid was reclassified as a morphologic subtype of anaplastic thyroid carcinoma (ATC). The squamous cell carcinoma pattern is similar to the other histologic types of ATC, but the phenotype associated has a poorer prognosis. The typical clinical manifestation of this condition is a cervical mass, accompanied by indications and symptoms of compression on adjacent structures such as the esophagus and trachea in advanced stages. Secondary squamous cell carcinoma of the thyroid may occur due to the spread of squamous carcinoma of the larynx or esophagus or distant metastases from other sites. Diagnosis of squamous cell carcinoma of the thyroid includes neck Ultrasound (US), Computed Tomography (CT) or Magnetic Resonance Imaging (MRI), puncture tissue biopsy, and full endoscopy to identify metastatic lesions from the nasopharynx, oropharynx, hypopharynx, larynx, esophagus, or bronchi and to help with the initial staging of the tumor. Current treatment modalities include surgery, radiotherapy, chemotherapy, or a combination. Because of the poor prognosis of patients with this disease, the short survival period, usually less than one year, and the difficulty of preoperative diagnosis, this article reviews the epidemiological features, origin, clinical features, pathological features, and differential diagnosis to improve the diagnosis and treatment of this disease by clinicians.

## 1 Introduction

Squamous cell carcinoma of the thyroid (SCCT) is a rare malignant tumor of the thyroid, including primary squamous cell carcinoma of the thyroid (PSCCT) and secondary squamous cell carcinoma of the thyroid (SSCCT). In 1988, the World Health Organization (WHO) classified squamous cell carcinoma of the thyroid as other cancers in the category of malignant epithelial neoplasms in the 2nd edition of the histologic typing of thyroid tumors. In the 3rd edition of the WHO classification of tumors, published in 2004, squamous cell carcinoma of the thyroid became a separate entity of thyroid tumors for the first time. In the 4th edition of the WHO classification of endocrine tumors, SCCT differs from thyroid cancer (1). However, in 2022, the 5th edition of the WHO classification of thyroid tumors classified it as a subtype of anaplastic thyroid carcinoma (ATC) (2–4).

SCCT is classified as a subtype of ATC for the following reasons (3). Firstly, their clinical manifestations are similar. Squamous cell carcinoma, with or without a differentiated thyroid cancer component, also has a prognosis comparable to ATC. Besides, molecular analysis shows ATC often has a BRAF V600E mutation associated with a history of papillary thyroid or other differentiated thyroid cancer (4, 5). At the same time, most SCCT carry the BRAF V600E mutation and exhibit TTF1 and PAX8 immune expression, demonstrating the origin of follicular cells. Studies have shown these SCCT expressed PAX8 (6) and TTF1 (7) in 91% and 38% of cases, respectively, confirming their follicular cell origin. Lam (8) found that half of SCCT cases had p53 overexpression. The frequency of overexpression was similar to that of ATC. BRAF, RAS, TERT promoter, and P53 were detected in 45%, 24%, 75%, and 63% of ATCs, respectively. In addition, SCCT can appear with well-differentiated thyroid cancers; pure squamous cell carcinomas not accompanied by differentiated thyroid carcinomas often have genetic alterations in the MAPK pathway that resemble thyroid follicular cell-derived carcinomas (8).

Early diagnosis is challenging due to the lack of clinical features, and it is also difficult to distinguish from other thyroid malignancies. The SCCT can develop extensive local infiltration and metastasis in the early stage. A common clinical feature in the late phase is tumor compression leading to compression of the esophagus and trachea, such as dysphagia and dyspnea. The course of the disease is very aggressive, and the prognosis is poor. There is no suitable treatment method for this disease, and the accepted treatment method is a comprehensive treatment based on surgical resection.

## 2 Epidemiology and incidence

SCCT is more common in older women, and most SCCT patients are about 50-60 years old (9, 10). The female-to-male ratio is 1.37-2.5 (11). The median age of ATC diagnosis is 68 years, and the female-to-male ratio is 1.2:1, comparable to that of SCCT. Because of the low incidence of the disease, primarily sporadic cases, its geographical distribution has not been studied. A review of 117 SCCT cases showed that 48% (n=56) of the cases were reported in Asia, most of which were reported in Japan. Other patients reported about the same in the United States (27%; n=32) (mainly in the United States) and Europe (25%; n=29) (mainly in the UK) (1). A study also concluded that geographical location was not a prognostic factor (10). PSCCT is an extremely rare thyroid malignancy, accounting for less than 1% of all thyroid malignancies (12). So far, only hundreds of cases have been reported, but some autopsy studies suggest that its incidence is not low, accounting for 28.4% of thyroid cancer. Possible reasons for the rarity of PSCCT in clinical practice are as follows:

(1) It is included in other types of thyroid cancer, such as poorly differentiated carcinoma;
(2) The patient dies without diagnosis or treatment and has a concise clinical course;
(3) It may manifest a different stage of thyroid cancer, such as papillary thyroid carcinoma transforming into squamous carcinoma that is diagnosed and treated.

SSCCTs occur in 1.2% to 24% of cases (13) and are rare because:

(1) The rapid flow of arterial blood prevents the adhesion of tumor cells (9, 14);
(2) The presence of iodine in the thyroid inhibits the proliferation of tumor cells (15, 16).

In contrast with PSCCT, SSCCT is a much rarer and more aggressive malignancy with a much lower incidence. Its diagnosis is complicated by the need to exclude other tumors, making SSCCT rarely diagnosed in the clinic for the first time. So, it is often clinically and pathologically misidentified as PSCCT (17).

## 3 Origin

Since the thyroid gland does not have a squamous epithelial component in its anatomy, the cause and the source of squamous cell carcinoma of the thyroid remain highly controversial. There are three theories of the origin of primary squamous cell carcinoma of the thyroid (10).

(1) Squamous epithelial metaplasia theory. The theory suggests that the thyroid gland is stimulated by various pathological conditions, such as inflammation, adenoma, papillary carcinoma, etc., and the follicular epithelial cells of the thyroid gland over-proliferate and develop squamous epithelial metaplasia, which can eventually lead to squamous cell carcinoma when the squamous cells continue to transform. Patients with SCCT may have a long history of hyperthyroidism and goiter. Pathology shows localized squamous epithelial hyperplasia, suggesting that benign thyroid lesions may also cause squamous epithelial hyperplasia. Giuseppe (18) reports a case of mixed squamous cell carcinoma and thyroid follicular carcinoma. Kallel (19) reported a case of SCCT coexisting with papillary thyroid carcinoma (PTC) and Hashimoto’s thyroiditis, whose pathology showed areas of squamous metaplasia in the region of chronic Hashimoto’s thyroiditis, while the hypercellular variant of papillary carcinoma was located away from the SCCT; therefore, the origin of SCCT in this patient was most likely a transformation of Hashimoto’s thyroiditis with an intermediate metaplasia stage.
(2) The residual embryonic theory. Goldberg and Harvey first proposed it (20). They suggested that during embryonic development, the residual squamous epithelial cells of the thyroglossal duct associated with the developing thyroid tissue metastasize into or on the surface of the glandular parenchyma and deteriorate into squamous cell carcinoma. During the fourth week of embryonic development, the thyroid initiation base (thyroglossal duct) descends and develops into the thyroid gland at the thyroid cartilage. This duct degenerates and disappears by the sixth week of embryonic development. If the degeneration of the thyroglossal duct is incomplete, the remaining epithelium may form a thyroglossal cyst (21). Some scholars analyzed the tissue of thyroglossal duct cysts in 88 patients and found that the epithelium of the cyst wall was squamous epithelial cells in 13 of them. However, some scholars have objected that the lowest part of the thyroglossal duct forms the thyroid gland’s cone lobe. If these squamous cells cause malignancy, then squamous cell carcinoma will appear in the cone lobe of the thyroid gland. However, most clinical cases of primary squamous cell carcinoma of the thyroid originate in the lateral lobes of the thyroid.
(3) Dedifferentiation of thyroid cancer. Primary squamous cell carcinoma of the thyroid may be a manifestation of different stages in thyroid adenocarcinoma (22). Chen Jun et al. (23) reported a patient with papillary carcinoma who was followed for ten years and underwent multiple surgeries. The postoperative pathology showed a progression from papillary carcinoma to papillary carcinoma with squamous transformation and then to squamous carcinoma. The progression from papillary carcinoma to squamous carcinoma was long, lasting nine years. In contrast, the progression from squamous cell metaplasia to squamous carcinoma was very rapid, lasting only one year, so it is assumed that squamous metaplasia can be considered a source of squamous thyroid carcinoma. Wiseman et al. found that (24) thyroid tumor cells can transform at the genetic level. For example, papillary carcinoma cells can transform into undifferentiated carcinoma cells. Alina Basnet et al. detected (25) mutations in the BRAF gene in a specimen associated with a patient with high cellular variant (TCV) papillary carcinoma that exhibited squamous transformation years later. All this evidence supports that squamous cell carcinoma of the thyroid originates from the dedifferentiation of other malignant thyroid tumors.

Genetic alterations in SCC include early and late molecular events, with early driver events mainly being RAS and BRAF mutations and RAS mutations associated with papillary carcinoma progression to hypo-fractionated carcinoma (26). Late changes mainly include TP53 and TERT promoter mutations, dysregulation of genes involved in the cell cycle, chromatin remodeling, histone modifications, and DNA mismatch repair (27). Morphologically, the development of SCC is also a multistep process. First, loss of heterozygosity (LOH), followed by loss of chromosome 3p and 9p21 regions, follows. This LOH and hypermethylation effectively inactivate the p16 gene, an inhibitor of cyclin-dependent kinases.

The above process allows a shift from typical squamous cell structure to histological atypia.

Second, an LOH at the 17p locus, accompanied by tumor suppressor gene p53 mutations, promotes cellular dysplasia. Finally, deletion of 4q, 6p, 8p, 11q, 13q and 14q eventually leads to overexpression of the cyclin D1 gene (located at 11q13). This activates the cell cycle process, ostensibly leading to the development of squamous cell carcinoma (28).

Secondary squamous cell carcinoma of the thyroid mostly comes from direct invasions of adjacent organs in the neck, such as the larynx, pharynx, esophagus, trachea, soft tissue, or mediastinum. Or from metastasis to other distant organs, such as the kidneys, uterus (29), gastrointestinal tract, colorectum, skin, bone marrow, prostate, and hematologic tumors. However, the cell type of SSCCT is more commonly seen as a direct extension of the larynx and pharynx (30). Therefore, in any patient with a known history of tumors, one should be alert to the potential risk of metastasis when a new thyroid mass is present. The location of the mass may provide a guide to the location of the primary tumor (15).

## 4 Clinical signs

Most patients present with neck swelling with or without hoarseness. Neck swelling is the most common complaint of patients, which may be caused by rapid tumor growth. Due to the highly aggressive nature of primary squamous cell carcinoma of the thyroid, rapidly enlarging neck masses invade the muscles, soft tissues, and blood vessels of the neck, resulting in neck pain (31). The tumor invades the trachea and esophagus, causing difficulty in breathing and swallowing, and compresses or invades the recurrent laryngeal nerve, causing hoarseness. Some patients also have cervical lymph node metastases.

Secondary squamous cell carcinoma of the thyroid gland is associated with other symptoms due to the primary tumor, such as cough and weight loss, in addition to the manifestations mentioned above. Once diagnosing metastasis, the clinician must look for the primary tumor.

The likely primary site can be determined by reviewing the patient’s medical history and physical examination, but a whole-body exam may also be required (15).

## 5 Diagnostics

Squamous cell carcinoma of the thyroid is highly aggressive and has a very different prognosis from common malignant tumors of the thyroid gland. Early and definitive diagnosis is essential to reduce the mortality rate of patients. The diagnosis requires a combination of clinical symptoms, imaging, pathology, and immunohistochemistry to confirm the diagnosis. Before diagnosing primary squamous cell carcinoma of the thyroid, secondary involvement of the thyroid, primary squamous cell carcinoma of adjacent structures, and metastases to the head and neck, chest, upper gastrointestinal tract, and pelvis must be excluded (32). A complete endoscopic examination should be performed to exclude primary lesions in the nasopharynx, oropharynx, hypopharynx, larynx, esophagus, or bronchi and to help with the initial staging of the tumor.

### 5.1 Laboratory tests

Laboratory tests are not specific for patients with squamous cell carcinoma of the thyroid. However, calcitonin can exclude medullary thyroid carcinoma to some extent. The thyroid function indexes may be thyrotoxicosis or hypothyroidism due to the different damage degree of metastasis to the thyroid gland. In the Ou (33) study, 8 out of 12 patients had higher than normal thyroglobulin levels. Three of the seven patients had higher than average levels of CA199, and three had higher than normal levels of squamous cell carcinoma antigen. As patients relapsed, squamous cell carcinoma antigen levels increased. The squamous cell carcinoma antigen level of 1 patient was standard at the first diagnosis. Still, it showed an elevated trend when it recurred, which may provide a new clinical indicator for postoperative review and recurrence detection of PSCTC patients.

### 5.2 Imaging examination

**Figure 1:**
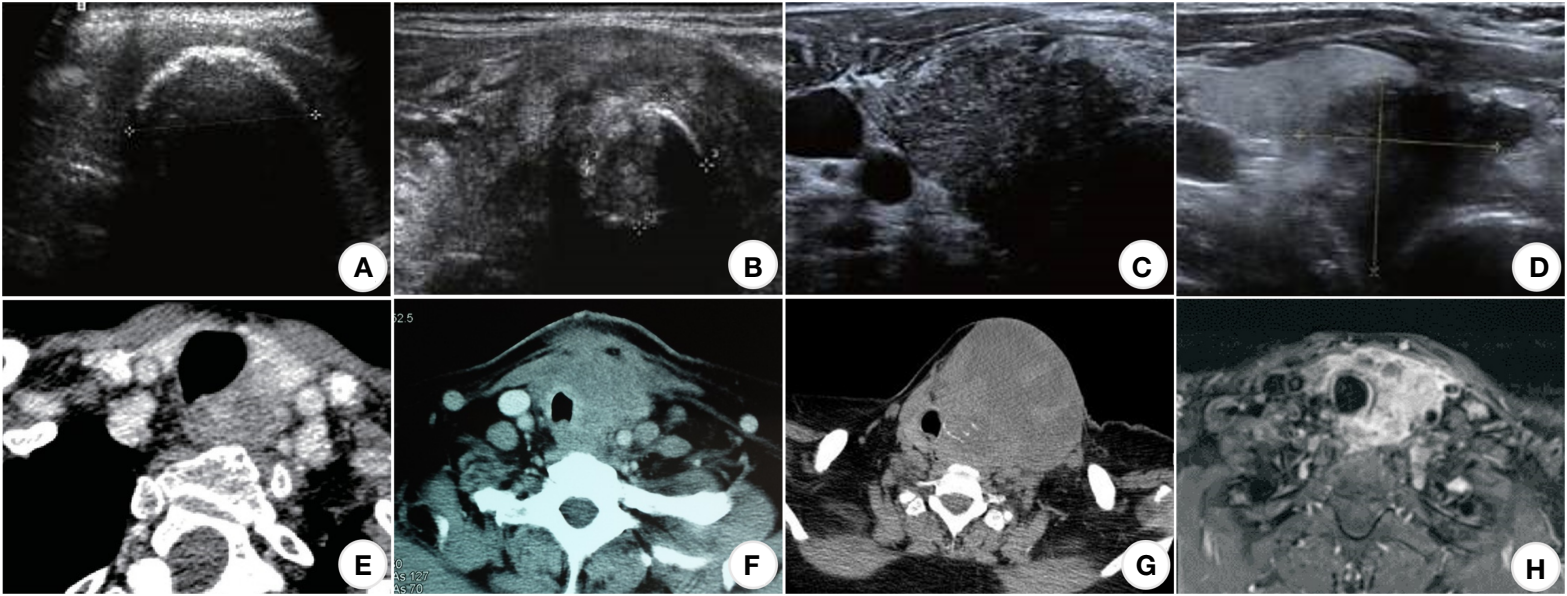
US, CT, and MRI images of SCCT. **(A)** (US) Eggshell-shaped calcification on the surface of the nodule; internal echoes are unavailable (33); **(B)** (US) Ring-shaped calcifications within the nodule (33); **(C)** (US) Punctate calcification within the nodule (33); **(D)** (US) Hypoechoic nodule with unclear borders, breaking through the thyroid peritoneum and invading the surrounding tissue (33); **(E)** (CT) Uneven moderate enhancement of the nodules, the esophagus and trachea were compressed and displaced, and the boundary was unclear; **(F)** (CT) The mass compresses and infiltrates the trachea and surrounding soft tissues (36); **(G)** (CT) Partial necrosis of the mass in the left lobe of the thyroid infiltrating adjacent skin and trachea (31); **(H)** (MRI) Uneven enhancement of the left lobe of the thyroid, with unclear boundaries with the esophagus, trachea, and left common carotid artery (37).

Ultrasonography is one of the preferred methods for clinical diagnosis of thyroid disorders. The test is simple and cost-effective, but because of the low incidence of squamous cell carcinoma of the thyroid, there are few clinical studies targeting ultrasound technology for diagnosing squamous cell carcinoma of the thyroid, which has no specific ultrasound imaging features. Its features are similar to other thyroid malignancies. Most of the nodules were large. A study (33) found that 48.5% of the tumors had a maximum diameter ≥50mm (13 cases in total), and the average tumor size was 51.5mm, which was consistent with the symptoms of the rapid growth of the neck mass accompanied by compression. The nodules have irregular shapes and unclear boundaries under ultrasound, all of which are solid mixed echoes of different degrees, mixed with a variety of high echo, shallow echo areas, and irregular vertical striation attenuation zones, indicating that the tumor growth rate is too fast, the occurrence of liquefaction and necrosis within the tumor or the aggregation of tumor cells (34, 35). Most approach or break through the thyroid envelope (**Figure 1D**). Regarding calcification, some patients had a few trace calcifications, eggshell-like calcifications, or discontinuous circular calcifications within the mass (38) (**Figures 1A–C**). The calcification may be dedifferentiated from differentiated thyroid carcinoma (papillary carcinoma) since fine-grained calcification is a characteristic manifestation of differentiated thyroid carcinoma (papillary carcinoma). Some patients do not show significant calcification. Regarding blood flow, the nodal Adler flow grading is concentrated in grades 2 to 3, and the flow index is between 0.7 and 0.9 (39). Approximately 20% of lesions in the SSCCT patients were scattered hyperechoic, and 40% of lesions had blood flow signals. Thyroid diffuse metastasis could also be seen in 7% of PSCCT patients and 20% of SSCCT patients.

CT and MRI are better than ultrasound for demonstrating thyroid tumors and their relationship to surrounding tissues and can show retrosternal lesions (40). It can also evaluate the adjacent larynx and trachea to identify migration, luminal narrowing, and invasion (41) (**Figures 1E–H**).

Computed tomography scans of the chest, abdomen, and pelvis are helpful to rule out secondary squamous cell carcinoma of the thyroid as the primary source. Primary squamous cell carcinoma of the thyroid usually tends to envelop the esophagus rather than invade it, leaving a fatty plane between the tumor and the esophagus, which helps to confirm that cancer originated in the thyroid rather than the esophagus itself (32, 42). Multiple nodules more often characterize the metastatic disease. In patients with a known primary tumor, this should raise a strong suspicion of metastatic disease.

### 5.3 Fine needle aspiration

Fine needle aspiration has proven to be a safe, minimally invasive, accurate, and cost-effective adjunctive diagnostic technique over the past few decades (43). The 2015 ATA guidelines for managing adults with thyroid nodules and differentiated thyroid cancer consider fine needle aspiration biopsy of the thyroid to be the most accurate and cost-effective method for evaluating thyroid nodules, with a recommendation level of solid recommendation and high-quality evidence (44).

Macroscopically, primary squamous cell carcinoma of the thyroid usually involves one or both lobes of the thyroid gland; in contrast, secondary squamous cell carcinoma of the thyroid is typically multifocal. Therefore, multiple punctures should be considered for characterization when metastatic malignant thyroid tumors are highly suspected, and repeat punctures may be an option when the results are inconclusive at one time (45, 46).

Although pathological diagnosis is the gold standard for the diagnosis of squamous thyroid cancer, it should be noted that needle aspiration cytology cannot be used as a standard for the diagnosis of primary squamous cell carcinoma of the thyroid for the following reasons:

(1) First, the needle prick tissue is small, and the cytologic features are challenging to identify metastatic squamous cell carcinoma and squamous metaplasia of papillary thyroid carcinoma.
(2) Second, as mentioned previously, primary squamous cell carcinoma of the thyroid may manifest in different periods in thyroid adenocarcinoma.
(3) Third, solid tumors have significant heterogeneity, such as temporal, spatial, and inter-tumor heterogeneity. The structural components of tumors are constantly changing, and the composition of different regions within the cancer varies.
(4) Fourth, it has been speculated that cancer cells may be firmly anchored within the tumor through fibrotic and interstitial proliferative responses and, therefore, not detected by FNA. Still, the prevalence of this phenomenon has yet to be determined (45, 47).

In cases where only fine-needle aspiration is performed, the diagnosis is often mistaken for other types of thyroid cancer, such as papillary carcinoma. It is difficult to make a definitive diagnosis because of the small number of cellular specimens obtained. Multiple punctures or coarse needle aspiration and immunohistochemistry are required to aid in the diagnosis. In a meta-analysis, Jae Keun Cho (48)concluded that the positive predictive value of FNAC for PSCCT may be less than 0.33 (28, 36, 45). and that FNAC is misdiagnosed as papillary thyroid carcinoma or non-diagnostic lesions in more than 50% of patients with PSCCT (15).

### 5.4 Pathological examination

**Figure 2:**
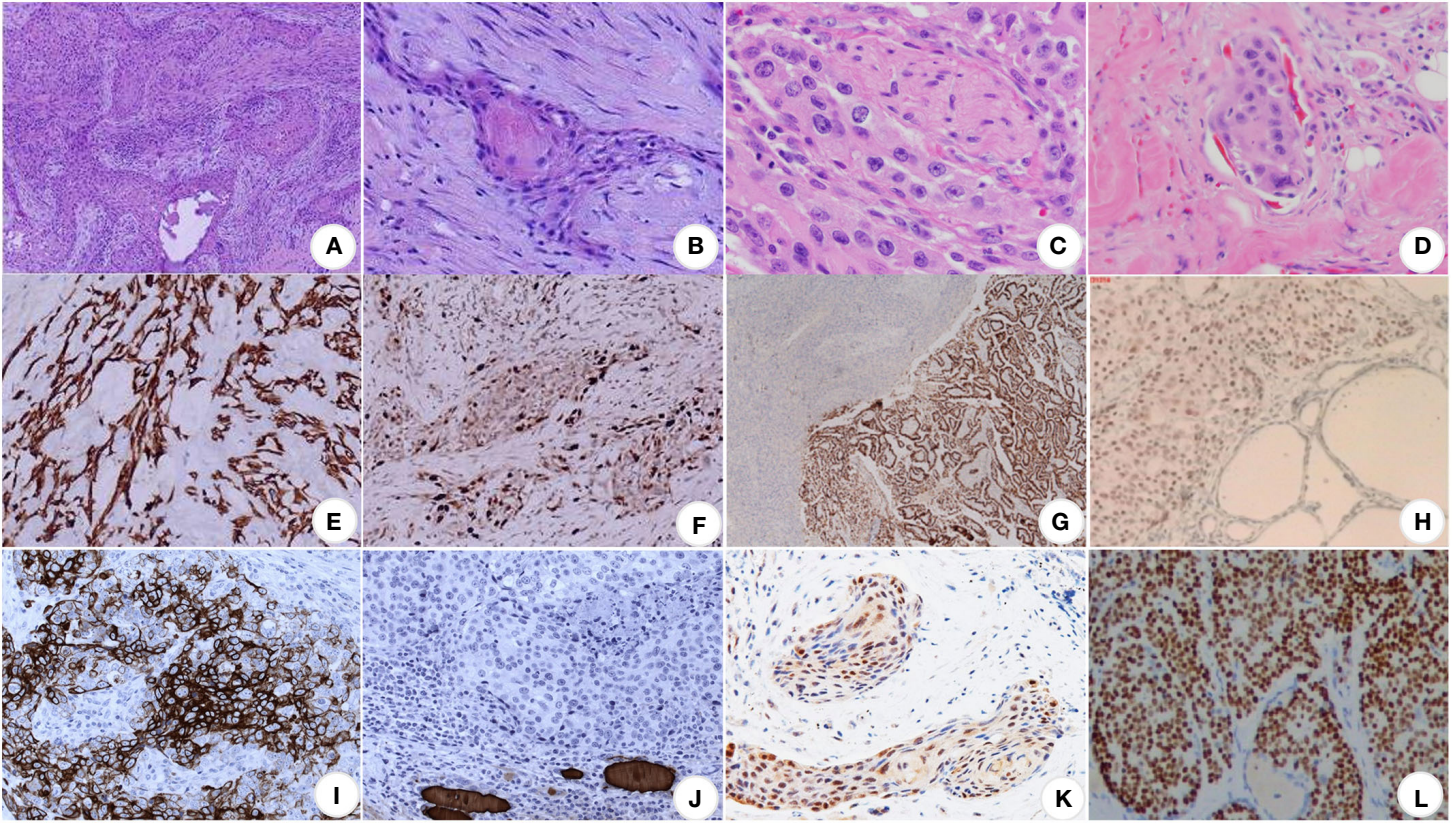
Pathological images of SCCT. **(A)** At low magnification, the cancerous tissues are in the form of striated, nested, and sheet-like structures, closely arranged, with deeply stained nuclei and numerous nuclear schizophrenic images, ×100 (9); **(B)** at high magnification, the formation of keratinized beads, highly stained nuclei, eosinophilic nucleoli and abundant eosinophilic cytoplasm can be seen, ×400 (9); **(C)** perineural invasion (50); **(D)** lymphovascular invasion (50); **(E)** CK19 (+) (9); **(F)** Ki-67 proliferation index of about 30% (9); **(G)** TTF1 (+) (16); **(H)** P63 (+) (51); **(I)** CK5/6 (+) (52); **(J)** TG (+) (52); **(K)** PAX8 is moderately positive (53); **(L)** p40 (+) (54).

Squamous cell carcinoma of the thyroid gland is locally nodular under the naked eye, with a hard texture, grayish-white cut surface, and partially fibrotic. Microscopically, the tumor cells were arranged in a nested pattern, and the typical morphological changes of squamous carcinomas, such as intercellular bridges, keratinized beads, and single-cell keratinization, could be seen (49) (**Figures 2A, B**). Peripheral nerves and lymphatic vessel infiltration are sometimes seen (**Figures 2C, D**). Primary thyroid gland tumors are easily distinguished from secondary tumors pathologically. If this is difficult, immunohistochemistry may be added.

Typical positive stains (28, 52) are keratin (especially CK7 and CK19), thyroglobulin, TTF-1, p53, and Ki-67; specific negative stains are CK20 and calcitonin (**Figures 2E–L**).

The expression pattern of cytokeratin differs in carcinomas of different organs and also varies with tumor differentiation (55, 56). CK19 has been reported to be strongly positive in primary squamous cell carcinoma of the thyroid but negative for CK1,4,10/13, and 20.

TTF-1 is a nuclear protein that regulates thyroid transcriptional activity. TTF-1 nuclear staining is positive in proliferative and neoplastic thyroid tissue, but immunoreactivity is less common in undifferentiated thyroid cancer. Focal TTF-1 and CK7 positivity support the possibility that the tumor cells are primary thyroid squamous cell carcinoma rather than extrathyroidal (e.g., laryngeal or oropharyngeal) origin. p53 is a 17p13 tumor suppressor gene and produces phosphorylated nuclear proteins involved in transcriptional regulation. The presence of p53 in 5% of nuclei is considered a positive result. p53 mutations are usually absent in highly differentiated thyroid cancers but are frequently seen in mesenchymal thyroid cancers (50 - 80%) and less regularly in poorly differentiated thyroid cancers. p53 overexpression is an indicator of poor differentiation (55). Raman spectroscopy was used to analyze TP53 gene mutations in tumor tissues of these patients, and the mutation frequency was as high as 42% (57). P63 is a transcription factor of the p53 gene family, and P63 has been widely used as a lineage marker for squamous cell carcinoma. Yan’s study showed 100% (4/4) positive expression (11). However, the sample size of this study is small, and the experiment can be generalized only after a large number of samples. The positive expression of p63 in thyroid tumors is not limited to squamous carcinoma. Tan (58) reported that p63 was expressed in 41.2% of thyroid papillary carcinoma, 28.6% of follicular carcinoma, and 66.7% of follicular adenoma.

Ki-67 is an unstable non-histone nuclear protein expressed in the cell cycle’s G1, S, G2, and M phases. It is rapidly catabolized at the M phase’s end and undetectable in the G0 and early G1 phases. Ki-67 is a cell proliferation marker interpreted as positive by the percent reactivity index.

Both p53 and Ki-67 staining at 30% supported tumor proliferation, not just squamous chemotaxis processes (28). The p53 and Ki-67 staining was associated with poorly differentiated thyroid cancer and risk of postoperative local recurrence and may also be a predictor of poor prognosis in squamous cell carcinoma of the thyroid.

PAX-8 is a transcription factor expressed in normal or tumor thyroid follicular epithelial cells and is present in 80% of mesenchymal thyroid cancers (ATCs). However, PAX-8 is rarely expressed in lung, pharyngeal, thymic, or cutaneous SCCs. Suzuki (53) et al. evaluated the expression of PAX-8 in a variety of thyroid adenocarcinomas (PTC, ATCs, mucinous epidermal carcinoma, PSSCCT) as well as uterine, esophageal, and lung SCCs. They found that 90.9% of thyroid SCCs expressed PAX-8, whereas extra-thyroidal sites of Vatsyayanet et al. studied 32 cases of head and neck SCC metastasizing to the thyroid. The histopathological features of these cases were similar to PSCCT but were harmful to PAX-8. Thus, PAX-8 (50) is a vital biomarker and has excellent value in differentiating primary squamous cell carcinoma of the thyroid from metastatic squamous cell carcinoma. Positive PAX-8 usually suggests PSCCT, whereas negative results suggest metastatic SCC from other sites (15, 53, 59).

### 5.5 Differential diagnosis

Undifferentiated thyroid carcinoma is expected in seniors, with high malignancy, rapid development, and aggressive tumor, and patients often have obvious invasive and compressive symptoms. The histomorphology consists of spindle, polymorphic giant, and epithelial-like cells, with apparent cell heterogeneity, nuclear schizophrenia, and necrosis. Immunohistochemical markers were negative for CD5 and positive for TG and TTF1. The tumor cells can express epithelial markers such as CK and EMA but not thyroid antigens such as TG.

Thymus-like differentiation carcinoma showing thymus-like differentiation (CASTLE) is a rare tumor with slow growth and a good prognosis (60). Thymus-like differentiation occurs in adults and offers a lobular growth pattern similar to lymphoepithelioma (61). PSCCT and CASTLE show central metaplasia with squamous differentiation and distinct cell borders. However, CASTLE stains positive for CD5, while PSCCT mainly does not express positivity (62).

## 6 Therapy

There is no acknowledged, accepted treatment for squamous thyroid cancer, and the generally accepted treatment plan is surgery followed by local radiotherapy and chemotherapy. Since most patients have locally advanced diseases at the time of discovery and radical resection is impossible, this disease emphasizes early detection, diagnosis, and treatment. Improving the examination at an early stage, assessing whether there is distant metastasis and airway obstruction, and performing adequate identification of whether it is metastasis in the case of neighboring organs considered to be tumors requires a complete examination, multidisciplinary discussion, and formulation of the individualized treatment plan (31).

Surgical treatment remains the primary treatment modality for this disease (63). In surgical treatment, a tiny percentage of patients have relatively small tumors at the time of discovery, and there is a chance of complete surgical resection of the tumor. However, because squamous cell carcinoma of the thyroid gland is highly infiltrative and has a deeper degree of infiltration, it is crucial to ensure the extent of surgical resection as much as possible and remove the primary foci and the invaded part of the metastases. Intraoperative freezing can more accurately determine whether the resection area is adequate. After statistical analysis of the clinical indicators, it was found that patients who underwent radical or extensive resection and postoperative local radiotherapy had a more prolonged survival. A tracheotomy may be performed first for patients with severe clinical symptoms and respiratory distress due to tracheal tumor compression. However, some scholars say that surgical resection should be limited to cases where complete surgical resection is possible (52).

If the outcome of surgical treatment is still not promising, if complete resection is difficult, or if peripheral tissue invasion occurs, postoperative radiotherapy should be used to improve the local control rate (64). Although it is generally believed that squamous thyroid carcinoma is not sensitive to radiotherapy (48), a growing body of literature affirms the role of postoperative radiotherapy. Inoperable patients may also benefit from radiotherapy. One patient who was not suitable for surgery, because the tumor encircled the left common carotid artery was treated with cisplatin-based concurrent radiotherapy and completed 47 days of treatment on schedule with significant results: the first CT scan at three months showed a considerable reduction of the mass (from 7 cm to 3.1 cm) and completed two years of disease-free survival (52).

Since squamous cell thyroid cancer itself is not sensitive to chemotherapy, most studies have shown that chemotherapy is not effective in squamous cell thyroid cancer. However, some scholars have proposed adjuvant chemotherapy (65). Adriamycin, bleomycin, cisplatin, 5-Fu, nitrogen mustard, vincristine, AB-132, adriamycin, or cyclophosphamide have been found to have little effect on this disease and have not shown a significant survival advantage (66–68).

Targeted therapies can be designed based on tumor genomics. For example, dabrafenib (BRAF inhibitor) and trametinib (MEK inhibitor) can be used in BRAFv600e-positive cases (69, 70), and laratinib can be used in NTRK- positive patients (15). Pembrolizumab is a highly selective monoclonal antibody, IgG4, against the cell surface programmed cell death protein-1 (PD-1) receptor (3). One study (71) reported a case of a patient with locally aggressive worsening of pulmonary metastases who had a progressive reduction in the size of the pulmonary metastases, but not the thyroid mass, during treatment with pembrolizumab.

Lenvatinib is a multi-receptor tyrosine kinase inhibitor that selectively inhibits multiple angiogenic and oncogenic signaling pathways. Recently, lenvatinib has been approved for treating radioiodine-refractory differentiated thyroid cancer in the United States and the European Union, and Japan for treating unresectable thyroid cancer, including interstitial thyroid cancer (ATC). Some studies have indicated its advantage in potentially prolonging survival (72). Still, it is essential to note that Lenvatinib has a risk of causing adverse bleeding events, especially in cases where the tumor is adjacent to important blood vessels (73). One study used Anlotinib + scintilla for PSCCT, and combining the two drugs reduced tumor volume rapidly (maximum diameter from 11.17 cm to 8.52 cm) (74). EGFR is a transmembrane tyrosine kinase receptor, which is overexpressed in PSCCT. Therefore, drugs targeting EGFR positivity are also a potential treatment modality for PSCCT patients (75).

The prognosis of patients is not affected by the use of thyroxine tablets after surgery. Oral supplementation with exogenous thyroxine suppresses thyroid cancer cells by feedback inhibition of thyrotropin (TSH) secretion from the pituitary gland. Since squamous cell carcinoma of the thyroid does not possess intracellular TSH receptors, TSH suppression therapy has little effect on this cancer type’s clinical course (76).

## 7 Future perspectives and conclusions

Compared with other differentiated thyroid cancers, squamous cell carcinoma of the thyroid is less prevalent. Still, it is more malignant, aggressive and progresses rapidly, and the prognosis is often poor. Patients die primarily due to local disease progression and respiratory damage, such as tumor invasion into the larynx leading to fatal respiratory obstruction (67), distant metastasis, or treatment complications (77).

One study analyzing the survival of 294 patients with primary squamous cell carcinoma of the thyroid found that the six-month survival rate was 55.5%, the one-year survival rate was 32.9%, the three-year survival rate was 9.9%, and the five-year survival rate was 6.4%, with a median survival of seven months. PSCCT is more aggressive than SSCCT. Primary squamous cell carcinoma of the thyroid tends to metastasize to the cervical lymph nodes and distantly to the lung, mediastinal lymph nodes, and liver (78). The median survival of PSCCT was 7.7 months.

Secondary squamous cell carcinoma of the thyroid depends mainly on the primary site. The clinical survival of patients with secondary thyroid tumors is short. Failure to diagnose and treat early is the main reason for the poor prognosis of these patients. Often, there is a multi-year latency period between the diagnosis of the primary tumor and the appearance of the secondary thyroid tumor. In a cohort of patients gathered by the Mayo Clinic, thyroid metastases were detected in 11 individuals more than a decade or two following the primary tumor (79). Therefore, a new thyroid mass in any patient with a known history of tumors should alert the patient to the potential risk of metastasis. Early diagnosis and treatment of secondary thyroid tumors may help prolong survival in some patients. **Table 1** lists the relationship between treatment modalities and survival time (in months) of SCCT patients reported in some literature.

**Table 1:** SCCT treatment and survival (performed and reported in limited studies in literature).

Reference	research methodology	number of cases	Age/Range	Gender	Tumor	Treatment	Follow-up time (mth)	Cause of death/ survival rate
**zhou** (80)	Retrospective Study	4	28-71	F (4)	PSCCT (3)	S (1) S/R (1) S/R/C (1)	4 13 26	Dyspnea Dyspnea Alive
					PSCCT+PTC (1)	S/R (1)	6	Dyspnea
**zhao** (9)	Case Report	1	63	F	PSCCT	S	7	Alive
		1	69	M	SSCCT From the esophagus	S	4	Alive
**Yasumatsu** (72)	Retrospective Study	10	53-77	M (3) F (7)	PSCCT (8) PSCCT +PTC (2)	S/R/C (7) S/R (2) S (1)	Median survival: 8	1 year survival rate: 22.7% 2-year survival rate: 0%
**Thewjitcharoen** (16)	Case Report	1	79	F	PSCCT +PTC	S/I/R	18	Alive/Neck metastasis
**Shrestha** (28)	Case Report	1	75	F	PSCCT	S/R	31	Dyspnea
**Sapalidis** (36)	Case Report	1	65	F	PSCCT	S/R/C	5	Tumor infiltration; Dyspnea
**SANCHEZ-SOSA** (61)	Case Report	1	13	F	PSCCT+ PTC	S	2	Alive
**Tunio** (31)	Case Report	1	54	F	PSCCT	R	3	Dyspnea
**Ibrahim** (78)	Case Report	1	74	F	PSCCT	S/R/C	1	Other causes (infection)
**Mercante** (18)	Case Report	1	67	M	PSCCT+ FPTC	S/I/C	24	Alive
**Bonetti** (75)	Case Report	1	66	F	PSCCT	S/C	12	Death
		1	83	F	PSCCT	S/C	4	Other causes (Cardiovascular diseases)
**Liu** (74)	Case Report	1	80	M	PSCCT	S/C	5	Other causes (Explosive liver failure)
**Lam** (1)	Retrospective Study	117	13-89 average 61	F (83) M (34)	PSCCT	–	Median survival: 8	88 cases died of the disease (109 cases) 2-year survival rate: 0%
**Ko** (60)	Case Report	1	87	M	PSCCT	S/R	11	Death
**Kallel** (19)	Case Report	1	54	F	PSCCT+PTC	S/I/R	24	Alive
**Lam** (8)	Case Report	3	56-82	F	PSCCT	S	Median survival: 3	Dyspnea
**Jang** (47)	Case Report	1	70	F	PSCCT	S	3	Alive/Cervical metastasis
**Iwamoto** (81)	Case Report	1	64	M	PSCCT	S/R/C	12	Alive
**Iwaki** (73)	Case Report	1	59	F	PSCCT	C	24	Alive
**Hsieh** (71)	Case Report	1	68	M	PSCCT	R/C	15	Death
**Dong** (76)	Case Report	1	62	F	PSCCT+PTC	S/I/R	20	Alive
**Rosario** (62)	Case Report	1	76	M	PSCCT	R/C	24	Alive

F, female; M, male; PSCC, primary squamous cell carcinoma of the thyroid; SSCCT, secondary squamous cell carcinoma of the thyroid; PTC, papillary thyroid cancer; mth, months; S, surgery; R, radiotherapy; C, chemotherapy; I, Iodine-131 therapy.

## Author contributions

WD: Writing – original draft, Funding acquisition. XG: Writing – original draft, Conceptualization, Methodology. XR: Formal analysis, Writing – review & editing.

## References

[1] Lam AK. Squamous cell carcinoma of thyroid: a unique type of cancer in World Health Organization Classification. Endocr Relat Cancer (2020) 27(6):R177–R92. doi: 10.1530/ERC-20-0045 32252028

[2] Baloch ZW Asa SL Barletta JA Ghossein RA Juhlin CC Jung CK. Overview of the 2022 WHO classification of thyroid neoplasms. Endocr Pathol (2022) 33(1):27–63. doi: 10.1007/s12022-022-09707-3 35288841

[3] Christofer Juhlin C Mete O Baloch ZW. The 2022 WHO classification of thyroid tumors: novel concepts in nomenclature and grading. Endocr Relat Cancer (2023) 30(2):1–10. doi: 10.1530/ERC-22-0293 36445235

[4] Gupta S Guo R Erickson LA. Anaplastic thyroid carcinoma, squamous cell carcinoma pattern. Mayo Clin Proc (2022) 97(8):1584–5. doi: 10.1016/j.mayocp.2022.06.019 35933145

[5] Chen TY Lorch JH Wong KS Barletta JA. Histological features of BRAF V600E-mutant anaplastic thyroid carcinoma. Histopathol (2020) 77(2):314–20. doi: 10.1111/his.14144 32428249

[6] Bishop JA Sharma R Westra WH. PAX8 immunostaining of anaplastic thyroid carcinoma: a reliable means of discerning thyroid origin for undifferentiated tumors of the head and neck. Hum Pathol (2011) 42(12):1873–7. doi: 10.1016/j.humpath.2011.02.004 21663937

[7] Nonaka D Tang Y Chiriboga L Rivera M Ghossein R. Diagnostic utility of thyroid transcription factors Pax8 and TTF-2 (FoxE1) in thyroid epithelial neoplasms. Mod Pathol (2008) 21(2):192–200. doi: 10.1038/modpathol.3801002 18084247

[8] Lam K-Y Lo C-Y Liu M-C. Primary squamous cell carcinoma of the thyroid gland: an entity with aggressive clinical behaviour and distinctive cytokeratin expression pro®les. Histopathol (2001) 39:279–86. doi: 10.1046/j.1365-2559.2001.01207.x 11532039

[9] Zhao X Hao P Tian J Sun J Chen D Cui Z. Primary and metastatic squamous cell carcinoma of the thyroid gland: Two case reports. Open Life Sci (2022) 17(1):1148–54. doi: 10.1515/biol-2022-0475 PMC9483828 36185404

[10] Yang S Li C Shi X Ma B Xu W Jiang H. Primary squamous cell carcinoma in the thyroid gland: A population-based analysis using the SEER database. World J Surg (2019) 43(5):1249–55. doi: 10.1007/s00268-019-04906-2 30719559

[11] Yan W Chen H Li J Zhou R Su J. Primary squamous cell carcinoma of thyroid gland: 11 case reports and a population-based study. World J Surg Oncol (2022) 20(1):352. doi: 10.1186/s12957-022-02814-9 36329478 PMC9632099

[12] Au JK Alonso J Kuan EC Arshi A St John MA. Primary squamous cell carcinoma of the thyroid: A population-based analysis. Otolaryngol Head Neck Surg (2017) 157(1):25–9. doi: 10.1177/0194599817698436 28397584

[13] Pastorello RG Saieg MA. Metastases to the thyroid: potential cytologic mimics of primary thyroid neoplasms. Arch Pathol Lab Med (2019) 143(3):394–9. doi: 10.5858/arpa.2017-0570-RS 30444438

[14] Cichon S Anielski R Konturek A Barczynski M Cichon W. Metastases to the thyroid gland: seventeen cases operated on in a single clinical center. Langenbecks Arch Surg (2006) 391(6):581–7. doi: 10.1007/s00423-006-0081-1 16983577

[15] Xin S Li W Yuan N Shen C Zhang D Chai S. Primary squamous cell carcinoma of the thyroid: a case report. J Int Med Res (2021) 49(4):1–8. doi: 10.1177/03000605211004702 PMC8040576 33827322

[16] Thewjitcharoen Y Krittiyawong S Butadej S Nakasatien S Polchart S Junyangdikul P. De-differentiation of papillary thyroid carcinoma into squamous cell carcinoma in an elderly patient: A case report. Med (Baltimore) (2020) 99(16):e19892. doi: 10.1097/MD.0000000000019892 PMC7440287 32312017

[17] Liu G Xu X Chen G Liu Z. Analysis of primary and secondary squamous cell carcinoma of the thyroid gland: a retrospective study. Gland Surg (2021) 10(2):559–66. doi: 10.21037/gs-20-628 PMC7944080 33708539

[18] Mercante G Marchesi A Covello R Dainese L Spriano G. Mixed squamous cell carcinoma and follicular carcinoma of the thyroid gland. Auris Nasus Larynx (2012) 39(3):310–3. doi: 10.1016/j.anl.2011.07.003 21855238

[19] Kallel S Kallel R Ayadi S Ghorbel A. Primary squamous cell carcinoma of the thyroid associated with papillary thyroid carcinoma and Hashimoto's thyroiditis. Eur Ann Otorhinolaryngol Head Neck Dis (2018) 135(4):291–3. doi: 10.1016/j.anorl.2018.05.012 29914738

[20] Goldberg HM Harvey P. Squamous-cell cysts of the thyroid with special reference to the aetiology of squamous epithelium in the human thyroid. Br J Surg (1956) 43(182):565–9. doi: 10.1002/bjs.18004318203 13342413

[21] Shah S Kadakia S Khorsandi A Andersen A Iacob C Shin E. Squamous cell carcinoma in a thyroglossal duct cyst: A case report with review of the literature. Am J Otolaryngol (2015) 36(3):460–2. doi: 10.1016/j.amjoto.2015.01.012 25697085

[22] Beninato T Kluijfhout WP Drake FT Khanafshar E Gosnell JE Shen WT. Squamous differentiation in papillary thyroid carcinoma: a rare feature of aggressive disease. J Surg Res (2018) 223:39–45. doi: 10.1016/j.jss.2017.10.023 29433884

[23] Jun C. Primary papillary thyroid carcinoma transformed into squamous carcinoma case report and literature review. J Pract Oncol (2012) 27:403–5. doi: 10.13267/j.cnki.syzlzz.2012.04.025

[24] Wiseman SM Hicks WL Jr. Rigual NR Winston JS Tan D Anderson GR. Anaplastic thyroid cancer evolved from papillary carcinoma. Arch Otolaryngol Head Neck Surg (2003) 129:96–100. doi: 10.1001/archotol.129.1.96 12525202

[25] Basnet A Pandita A Fullmer J Sivapiragasam A. Squamous cell carcinoma of the thyroid as a result of anaplastic transformation from BRAF-positive papillary thyroid cancer. Case Rep Oncol Med (2017) 2017:4276435. doi: 10.1155/2017/4276435 29158933 PMC5660805

[26] Gopal PP Montone KT Baloch Z Tuluc M LiVolsi V. The variable presentations of anaplastic spindle cell squamous carcinoma associated with tall cell variant of papillary thyroid carcinoma. Thyroid (2011) 21(5):493–9. doi: 10.1089/thy.2010.0338 21309723

[27] Volante M Lam AK Papotti M Tallini G. Molecular pathology of poorly differentiated and anaplastic thyroid cancer: what do pathologists need to know? Endocr Pathol (2021) 32(1):63–76. doi: 10.1007/s12022-021-09665-2 33543394 PMC7960587

[28] Shrestha M Sridhara SK Leo LJ Coppit GL 3rd Ehrhardt NM. Primary squamous cell carcinoma of the thyroid gland: a case report and review. Head Neck (2013) 35(10):E299–303. doi: 10.1002/hed.23152 23002023

[29] Simon C Grunenwald S Soule-Tholy M Escourrou G Vezzosi D Deslandres M. Synchronous intrathyroid metastasis from undifferentiated endometrial sarcoma. Thyroid (2013) 23(7):902–3. doi: 10.1089/thy.2012.0594 23360518

[30] Nakhjavani M Gharib H Goellner JR Heerden JA. Direct extension of Malignant lesions to the thyroid gland from adjacent organs: report of 17 cases. Endocr Pract (1999) 5(2):69–71. doi: 10.4158/EP.5.2.69 15251691

[31] Tunio MA Al Asiri M Fagih M Akasha R. Primary squamous cell carcinoma of thyroid: a case report and review of literature. Head Neck Oncol (2012) 4:4–8. doi: 10.1186/1758-3284-4-8 22452749 PMC3331844

[32] Syed MI Stewart M Syed S Dahill S Adams C McLellan DR. Squamous cell carcinoma of the thyroid gland: primary or secondary disease? J Laryngol Otol (2011) 125(1):3–9. doi: 10.1017/S0022215110002070 20950510

[33] Ou D Ni C Yao J Lai M Chen C Zhang Y. Clinical analysis of 13 cases of primary squamous-cell thyroid carcinoma. Front Oncol (2022) 12:956289. doi: 10.3389/fonc.2022.956289 36052269 PMC9424675

[34] Chunlan H. Clinical study of ultrasonography in the diagnosis of primary thyroid squamous cell carcinoma. Imaging Res Med Appl (2002) 6(14):188–90.

[35] Wang Chunyan WX Pengchao ZHENG. Ultrasonographic features of primary squamous cell carcinoma of the thyroid gland. J China Med Univ (2020) 11(1051):3.

[36] Sapalidis K Anastasiadis I Panteli N Strati TM Liavas L Poulios C. Primary squamous cell carcinoma of the thyroid gland. J Surg Case Rep (2014) 2014(12):1–3. doi: 10.1093/jscr/rju133 PMC4258703 25487371

[37] Hu C Zhang X Jie D Zhang C Bai Z. Common carotid artery rupture after primary thyroid squamous cell carcinoma. Clin J Otolaryngol Head Neck Surg (2019) 35(12):1132–5. doi: 10.13201/j.isn.2096-7993.2021.12.016 PMC10127647 34886631

[38] Chen C-Y Tseng H-S Lee C-H Chan WP. Primary squamous cell carcinoma of the thyroid gland with eggshell calcification: sonographic and computed tomographic findings. J Ultrasound Med (2010) 29:1667–70. doi: 10.7863/jum.2010.29.11.1667 20966481

[39] Chen S Peng Q Zhang Q Niu C. Contrast-enhanced ultrasound of primary squamous cell carcinoma of the thyroid: A case report. Front Endocrinol (Lausanne) (2020) 11:512. doi: 10.3389/fendo.2020.00512 32849297 PMC7431615

[40] Gaillardin L Beutter P Cottier JP Arbion F Moriniere S. Thyroid gland invasion in laryngopharyngeal squamous cell carcinoma: prevalence, endoscopic and CT predictors. Eur Ann Otorhinolaryngol Head Neck Dis (2012) 129(1):1–5. doi: 10.1016/j.anorl.2011.04.002 21798840

[41] Kinshuck AJ Goodyear PW Lancaster J Roland NJ Jackson S Hanlon R. Accuracy of magnetic resonance imaging in diagnosing thyroid cartilage and thyroid gland invasion by squamous cell carcinoma in laryngectomy patients. J Laryngol Otol (2012) 126(3):302–6. doi: 10.1017/S0022215111003331 22234175

[42] Chu MMH Mirza O Bishop PW Pothula V. Primary squamous cell carcinoma of the thyroid gland successfully treated with surgical resection and adjuvant chemoradiotherapy. BMJ Case Rep (2021) 14(3):1–4. doi: 10.1136/bcr-2020-241209 PMC7929853 33649031

[43] Paschke R Hegedus L Alexander E Valcavi R Papini E Gharib H. Thyroid nodule guidelines: agreement, disagreement and need for future research. Nat Rev Endocrinol (2011) 7(6):354–61. doi: 10.1038/nrendo.2011.1 21364517

[44] Haugen BR Alexander EK Bible KC Doherty GM Mandel SJ Nikiforov YE. 2015 American thyroid association management guidelines for adult patients with thyroid nodules and differentiated thyroid cancer: the American thyroid association guidelines task force on thyroid nodules and differentiated thyroid cancer. Thyroid (2016) 26(1):1–133. doi: 10.1089/thy.2015.0020 26462967 PMC4739132

[45] Lichiardopol C Şurlin V Foarfă MC Ghiluşi MC Bondari S. Primary squamous cell carcinoma of the thyroid: a case report. Rom J Morphol Embryol (2016) 57(2 Suppl):831–6. 27833978

[46] Chen TLN H Udelsman R. Clinically significant, isolated metastatic disease to the thyroid gland. World J Surg (1999) 23:177–81. doi: 10.1007/PL00013162 9880428

[47] Jang JY Kwon KW Kim SW Youn I. Primary squamous cell carcinoma of thyroid gland with local recurrence: ultrasonographic and computed tomographic findings. Ultrasonography (2014) 33(2):143–8. doi: 10.14366/usg.13022 PMC4058984 24936508

[48] Cho JK Woo SH Park J Kim MJ Jeong HS. Primary squamous cell carcinomas in the thyroid gland: an individual participant data meta-analysis. Cancer Med (2014) 3(5):1396–403. doi: 10.1002/cam4.287 PMC4302690 24995699

[49] Wang K Shi N Yu M Zhou W Wang X Wang C. Anaplastic spindle cell squamous carcinoma arising from classical papillary thyroid carcinoma with foci of columnar cell component: A case report. Tissue Cell (2021) 73:101666. doi: 10.1016/j.tice.2021.101666 34678532

[50] Torrez M Braunberger RC Yilmaz E Agarwal S. Primary squamous cell carcinoma of thyroid with a novel BRAF mutation and High PDL-1 expression: A case report with treatment implications and review of literature. Pathol Res Pract (2020) 216(10):153146. doi: 10.1016/j.prp.2020.153146 32853962

[51] Rong WD. Pathological analysis and literature review of primary thyroid squamous cell carcinoma: a case report. J Qiqihar Med College (2014) 35(20):3014–5.

[52] Struller F Senne M Falch C Kirschniak A Konigsrainer A Muller S. Primary squamous cell carcinoma of the thyroid: Case report and systematic review of the literature. Int J Surg Case Rep (2017) 37:36–40. doi: 10.1016/j.ijscr.2017.06.011 28633125 PMC5479948

[53] Suzuki A Hirokawa M Takada N Higuchi M Yamao N Kuma S. Diagnostic significance of PAX8 in thyroid squamous cell carcinoma. Endocr J (2015) 62(11):991–5. doi: 10.1507/endocrj.EJ15-0226 26354716

[54] Huanfen D. A clinicopathological observation of metastatic esophageal squamous cell carcinoma manifested as thyroid nodule in 1 case. J Diagn Pathol (2021) 28(2):98–100.

[55] Lam KY Lo CY Liu MC. Primary squamous cell carcinoma of the thyroid gland: an entity with aggressive clinical behaviour and distinctive cytokeratin expression profiles. Histopathol (2001) 39(3):279–86. doi: 10.1046/j.1365-2559.2001.01207.x 11532039

[56] Fassan M Pennelli G Pelizzo MR Rugge M. Primary squamous cell carcinoma of the thyroid: immunohistochemical profile and literature review. Tumori (2007) 93(5):518–21. doi: 10.1177/030089160709300522 18038891

[57] Wang SS Ye DX Wang B Xie C. The expressions of keratins and P63 in primary squamous cell carcinoma of the thyroid gland: an application of Raman spectroscopy. Onco Targets Ther (2020) 13:585–91. doi: 10.2147/OTT.S229436 PMC6980845 32021300

[58] Tan A Etit D Bayol U Altinel D Tan S. Comparison of proliferating cell nuclear antigen, thyroid transcription factor-1, Ki-67, p63, p53 and high-molecular weight cytokeratin expressions in papillary thyroid carcinoma, follicular carcinoma, and follicular adenoma. Ann Diagn Pathol (2011) 15(2):108–16. doi: 10.1016/j.anndiagpath.2010.11.005 21315633

[59] Yasuoka H Nakamura Y Yoshida KI Shimo T Tori M Matsui Y. A rare case of ectopic papillary thyroid carcinoma transformed into squamous cell carcinoma. Pathol Int (2018) 68(4):246–50. doi: 10.1111/pin.12649 29451347

[60] Ko YS Hwang TS Han HS Lim SD Kim WS Oh SY. Primary pure squamous cell carcinoma of the thyroid: report and histogenic consideration of a case involving a BRAF mutation. Pathol Int (2012) 62(1):43–8. doi: 10.1111/j.1440-1827.2011.02745.x 22192803

[61] Sanchez-Sosa S Rios-Luna NP Tamayo Bdel R Simpson K Albores-Saavedra J. Primary squamous cell carcinoma of the thyroid arising in Hashimoto's thyroiditis in an adolescent. Pediatr Dev Pathol (2006) 9(6):496–500. doi: 10.2350/06-04-0073.1 17163793

[62] Del Rosario M Dasanu C Tsai H Johnson R. Primary squamous cell carcinoma of the thyroid with complete response to radical radiotherapy and concurrent cisplatin-based chemotherapy. BMJ Case Rep (2017) 2017:1–5. doi: 10.1136/bcr-2016-217143 PMC5256516 28100571

[63] Jin S Liu X Peng D Li D Ye YN. Differences between cancer-specific survival of patients with anaplastic and primary squamous cell thyroid carcinoma and factors influencing prognosis: A SEER database analysis. Front Endocrinol (Lausanne) (2022) 13:830760. doi: 10.3389/fendo.2022.830760 35360080 PMC8960140

[64] Ab Hadi I Bliss RD Lennard TWJ Welch AR. Primary squamous cell carcinoma of the thyroid gland: A case report and role of radiotherapy. Surgeon. (2007) 5(4):249–51. doi: 10.1016/s1479-666x(07)80010-2 17849961

[65] John Simpson W Carruthers J. Squamous cell carcinoma of the thyroid gland. Am J Surg. (1988) 156(1):44–6. doi: 10.1016/s0002-9610(88)80169-7 3394892

[66] Segal K Sidi J Abraham A Konichezky M Ben-Bassat M. Pure squamous cell carcinoma and mixed adenosquamous cell carcinoma of the thyroid gland. Head Neck Surg (1984) 6(6):1035–42. doi: 10.1002/hed.2890060610 6469655

[67] Booya F Sebo TJ Kasperbauer JL Fatourechi V. Primary squamous cell carcinoma of the thyroid: report of ten cases. THYROID (2006) 16:89–93. doi: 10.1089/thy.2006.16.89 16487020

[68] Cook AM Vini L Harmer C. Squamous cell carcinoma of the thyroid: outcome of treatment in 16 patients. Eur J Surg Oncol (1999) 25(6):606–9. doi: 10.1053/ejso.1999.0715 10556008

[69] Xu B Fuchs T Dogan S Landa I Katabi N Fagin JA. Dissecting anaplastic thyroid carcinoma: A comprehensive clinical, histologic, immunophenotypic, and molecular study of 360 cases. Thyroid (2020) 30(10):1505–17. doi: 10.1089/thy.2020.0086 PMC7583343 32284020

[70] Brandenburg T Muchalla P Theurer S Schmid KW Fuhrer D. Therapeutic effect of combined dabrafenib and trametinib treatment of BRAF V600E-mutated primary squamous cell carcinoma of the thyroid: A case report. Eur Thyroid J (2021) 10(6):511–6. doi: 10.1159/000518055 PMC8647138 34956922

[71] Hsieh ML Besch BM Peterson JEG Henson C. Primary squamous cell carcinoma of the thyroid treated with concurrent chemoradiation and palliative immunotherapy: a case report. J Med Case Rep (2022) 16(1):364. doi: 10.1186/s13256-022-03596-0 36195921 PMC9533597

[72] Yasumatsu R Sato M Uchi R Nakano T Hashimoto K Kogo R. The treatment and outcome analysis of primary squamous cell carcinoma of the thyroid. Auris Nasus Larynx (2018) 45(3):553–7. doi: 10.1016/j.anl.2017.07.009 28739190

[73] Iwaki S Kawakita D Sawabe M Matoba T Takano G Oguri K. Long-term efficacy of weekly paclitaxel therapy in unresectable primary squamous cell carcinoma of the thyroid. Auris Nasus Larynx (2022) 49(6):1083–7. doi: 10.1016/j.anl.2021.06.005 34226099

[74] Liu Z Yu M Zhao F Zhu C. Anlotinib combined with Sintilimab is win-win cooperation for primary squamous cell carcinoma of the thyroid: A case report and literature review. Front Oncol (2023) 13:976415. doi: 10.3389/fonc.2023.976415 37007162 PMC10062477

[75] Bonetti LR Lupi M Trani M Trani N Sartori G Schirosi L. EGFR polysomy in squamous cell carcinoma of the thyroid. Report of two cases and review of the literature. Tumori (2010) 96:5503–507. doi: 10.1177/030089161009600323 20845818

[76] Dong S Song XS Chen G Liu J. Mixed primary squamous cell carcinoma, follicular carcinoma, and micropapillary carcinoma of the thyroid gland: A case report. Auris Nasus Larynx (2016) 43(4):455–9. doi: 10.1016/j.anl.2015.10.011 26589365

[77] Agilinko J Kueh TJ Smart L Shakeel M. Primary thyroid squamous cell carcinoma: a challenging management problem. BMJ Case Rep (2021) 14(1):1–6. doi: 10.1136/bcr-2020-238560 PMC7845689 33509876

[78] Ibrahim M-I Jusoh Y-R Adam N-N Mohamad I. Primary squamous cell carcinoma of the thyroid gland. Iran J Otorhinolaryngol (2018) 30:65–8. PMC5787658 29387667

[79] Gharib H Papini E Paschke R. Thyroid nodules: a review of current guidelines, practices, and prospects. Eur J Endocrinol (2008) 159(5):493–505. doi: 10.1530/EJE-08-0135 18728120

[80] Zhou XH. Primary squamous cell carcinoma of the thyroid. Eur J Surg Oncol (2002) 28(1):42–5. doi: 10.1053/ejso.2001.1180 11869012

[81] Iwamoto Y Anno T Koyama K Ota Y Nakashima K Monobe Y. Primary squamous cell carcinoma of the thyroid with severe tracheal invasion: A case report. Eur Thyroid J (2021) 10(6):548–9. doi: 10.1159/000511709 PMC8647117 34950602

